# Supplementary material for: New Quinoxaline-Based Derivatives as PARP-1 Inhibitors: Design, Synthesis, Antiproliferative, and Computational Studies
Source: Molecules. 2022 Aug 2;27(15):4924. doi: 10.3390/molecules27154924 (PMC9370283; doi:10.3390/molecules27154924)
Supplement: Supplementary file 1 [file molecules-27-04924-s001.zip › molecules-1805532-supplementary.pdf]

Supplementary material

4. Experimental

4.1. Chemistry

All melting points are uncorrected and were taken in open capillary tubes using Electrothermal apparatus 9100. Elemental microanalyses were carried out at Microanalytical Unit, Central Services Laboratory, National Research Centre, Dokki, Cairo, Egypt, using Vario Elementar and were found within ±0.4% of the theoretical values. Infrared spectra were recorded on a Shimadzu FT-IR Affinity-1 Spectrometer, Infrared spectrometer at cm^{-1} scale using KBr disc technique at Faculty of Pharmacy/Cairo University/Cairo/ Egypt. ^{1}H NMR and ^{13}C NMR spectra were determined by using a Bruker High-Performance Digital FT-NMR Spectrometer Avance III 400MHz, Faculty of Pharmacy/Cairo University/Cairo/Egypt and at Chemistry Department, Prairie View A&M University. Chemical shifts were expressed in δ (ppm) downfield from TMS as an internal standard while the coupling constants (J values) are given in Hz. Follow up of the reactions and checking the purity of the compounds were made by TLC on silica gel-precoated aluminum sheets (Type 60, F 254, Merck, Darmstadt, Germany) using chloroform/methanol (10:0.8, v/v) and the spots were detected by exposure to UV lamp at δ 254 nanometre for few seconds and by iodine vapor. The chemical names given for the prepared compounds are according to the IUPAC system.

4.2. PARP-1 inhibition assay

PARP-1 enzyme inhibition activity was measured using a colorimetric 96-well PARP-1 assay kit (catalog no. 80580) (BPS Bioscience), according to the manufacturer's protocol. Briefly, the histone mixture was diluted 1: 5 with 1x PBS, and 50 µL of histone solution was added to each well and incubated at 4 °C overnight. The plate was washed three times using 200 µL PBST buffer (1x PBS containing 0.05% Tween-20) per well. The liquid was removed from the wells by tapping the strip wells on clean paper towels. To each well, 200 µL of blocking buffer was added, followed by 60–90 min incubation at room temperature. Then, 25 µL of PARP master mixture (consisting of 2.5 µL 10x PARP buffer + 2.5 µL 10x PARP assay mixture + 5 µL activated DNA + 15 µL distilled water) was added to each well. Olaparib was used as a positive control. 5 µL of inhibitor solution was added to each well labeled as "Test Inhibitor". For the "Positive Control" and "Blank", 5 µL of the same solution without inhibitor was added. 1x PARP buffer was prepared by adding 1 part of 10 x PARP buffer to 9 parts H_2O (v/v), and 20 µL of 1 x PARP buffer was added

to the wells designated as “Blank”. The amount of PARP-1 required for the assay was then calculated. The reaction was initiated by adding 20 µL of diluted PARP-1 enzyme to the wells designated “Positive Control” and “Test Inhibitor Control”. The strip wells were incubated at room temperature for 1 hour. The strip wells were then washed three times with 200 µL of PBST buffer. Then, 50 µL of 50 times diluted Streptavidin-HRP with blocking buffer was added to each well, and the strips were further incubated at room temperature for 30 min. After washing the wells three times with 200 µL of PBST buffer, HRP colorimetric substrate was added to each well, and the plate was incubated at room temperature until a blue color was developed in the positive control well. Then, the reaction was quenched with 100 µL per well of 2 M sulfuric acid. Carrier solvent (DMSO 5%, v/v) was used as a negative control. All compounds including Olaparib were tested at 5 concentrations (100, 10, 1, 0.1, and 0.01 µM). The optical density (OD) of each well was measured spectrophotometrically at 450 nm with an ELISA microplate reader (ChroMate-4300, FL, USA). The IC_{50} values were calculated according to the equation for Boltzmann sigmoidal concentration-response curves using the nonlinear regression fitting model (GraphPad Prism Version 8). All assays were performed in triplicate in 3 repeated experiments. The obtained data are presented as means standard error of the means from 3 independent repeats (n = 3).

4.3.*In vitro* anticancer screening

The cell lines were purchased from the American Type Culture collection as follows: Breast Cancer cell lines (MDA-MB-436) and WI38. Cytotoxic activity screening was performed using MTT assay. Exponentially, cells were placed in 10^4 cells/ well for 24 h, and then add fresh medium containing different concentrations of the tested sample. Serial two-fold dilutions of the tested sample were added using a multichannel pipette. Moreover, all cells were cultivated at 37 °C, with 5% CO_2 and 95% humidity. Also, incubation of control cells occurred at 37 °C. However, after incubation for 24 h different concentrations of the sample (50, 25, 12.5, 6.25, 3.125, 1.56, and 0 µg L^{-1}) were added and continued the incubation for 48 h, then, add the crystal violet solution 1% to each well for 0.5 h to examine viable cells. Rinse the wells using water until no stain. After that, add 30% glacial acetic acid to all wells with shaking plates on a Microplate reader (TECAN, Inc.) to measure the absorbance, using a test wavelength of 490 nm. Besides, compare the treated samples with the control cell. The cytotoxicity was estimated by IC_{50} in (µg /mL), the concentration that inhibits 50% of the growth of the examined cell.

4.4. Cell cycle analysis

The pre-calculated IC_{50} of compound **5** was applied to MDA-MB-436 breast cancer cells for 48h. The cells were treated with trypsin, rinsed two times in PBS, fixed in ice-cold 60% ethanol at 40 °C, and washed again in PBS. The cells are then re-suspended in 500 µL of propidium iodide (PI) with RNase staining buffer from "BD Pharmingen" in the United States and incubated for thirty minutes. Finally, FACS studies were performed using an ACEA Novocyte TM flow cytometer from ACEA Biosciences Inc. in San Diego, California. "ACEA Novo Express TM software, ACEA Biosciences Inc., San Diego, USA," was used to compile data from 12,000 cells for each sample and examine the distribution of cell cycle stages.

4.5. Apoptosis analysis

MDA-MB-436 cells were treated with compound 5 for 48h, then treated with trypsin and rinsed twice in PBS. Apoptosis assessment was done via the "Annexin V-FITC/PI Apoptosis Detection Kit", "BD Biosciences, San Diego, USA", as stated by the manufacturer. In a summary, cells were suspended again in 0.5 ml binding buffer, then 5 µL "Annexin V-FITC" and 5 µL PI (staining solution) were added for 30 min at ambient conditions in a dark location. Finally, the cells were implemented to "FACS analysis using ACEA Novocyte™ flow cytometer", "ACEA Biosciences Inc., San Diego, CA, USA", within one hour following staining.

4.6. Autophagy Analysis

To further confirm the cell death mechanism induced by the drugs, autophagic cell death was quantitatively analyzed using a Cyto-ID Autophagy Detection Kit (Abcam Inc., Cambridge Science Park, Cambridge, UK). In brief, cells were treated for 24 h by the IC_{50} value of the tested compound. Chloroquine treatment (10 µM) was used as a positive control, while a drug-free medium was used as a negative control. Cells were then washed twice with PBS and stained with Cyto-ID Green in the dark at 37 °C for 30 min according to the manufacturer's protocol. After staining, cells were analyzed for Cyto-ID differential green/orange fluorescent signals using an FL2 signal detector (λex/em 535/617 nm). Mean net fluorescent intensities (NFI) were quantified.

4.7. Docking methodology

The crustal structure of Olaparib in complex with the catalytic domain of PARP1 [42] was downloaded from the protein databank (www.rcsb.org). The complex structure was processed with the Protein Preparation Wizard [43,44] in Maestro [45] to add missing atoms, sidechains, and

residues, complete loops, add hydrogen atoms and adjust bond orders for amino acids and ligands. The protonation and tautomerization states were adjusted for a pH of 7.4. Water molecules within 5 Å to the ligand were kept if form at least three hydrogen bonds to the non-water residue. The hydrogen bond network was sampled at pH of 7.4, and the complex was subjected to thoroughly restrained minimization using OPLS3e force-field.

The receptor grid was prepared for the docking step using OPLS3e force-field by selecting olaparib coordinates as the center of the binding pocket. In order to add structural flexibility during the docking step, the Van der Waals radii were scaled by a factor of 1.0 to soften the potential of non-polar atoms of the target.

The chemical structures of the ligands were sketched in Maestro. The 3D conformers were constructed using OPLS3e force-field. Ligprep [46] was used to prepare the ligands to generate all possible protonation and ionization states at a pH of 7.4. The lowest energy conformer was kept for each ligand. Glide with SP docking precision (46-50) was used to dock the prepared ligands into PARP1's receptor grid. The softening potential was considered during the docking experiment with the default values. The top three docking poses were kept for the Prime/MM–GBSA method (10-12) to calculate the binding free energy (ΔGbind) of each ligand.

Spectral data

Figure Captions

Figure S1. ^{1}H NMR spectrum of compound **4**
Figure S2. ^{1}H NMR spectrum of compound **5**
Figure S3. ^{13}C NMR spectrum of compound **5**
Figure S4. ^{1}H NMR spectrum of compound **6**
Figure S5. ^{13}C NMR spectrum of compound **6**
Figure S6. ^{1}H NMR spectrum of compound **7c**
Figure S7. ^{13}C NMR spectrum of compound **7c**
Figure S8. ^{1}H NMR spectrum of compound **8a**
Figure S9. ^{13}C NMR spectrum of compound **8a**
Figure S10. ^{1}H NMR spectrum of compound **9a**
Figure S11. ^{1}H NMR spectrum of compound **9b**
Figure S12. ^{13}C NMR spectrum of compound **9b**
Figure S13. ^{1}H NMR spectrum of compound **10b**
Figure S14. ^{13}C NMR spectrum of compound **10b**
Figure S15. ^{1}H NMR spectrum of compound **11a**
Figure S16. ^{13}C NMR spectrum of compound **11a**
Figure S17. ^{1}H NMR spectrum of compound **11b**
Figure S18. ^{1}H NMR spectrum of compound **12a**

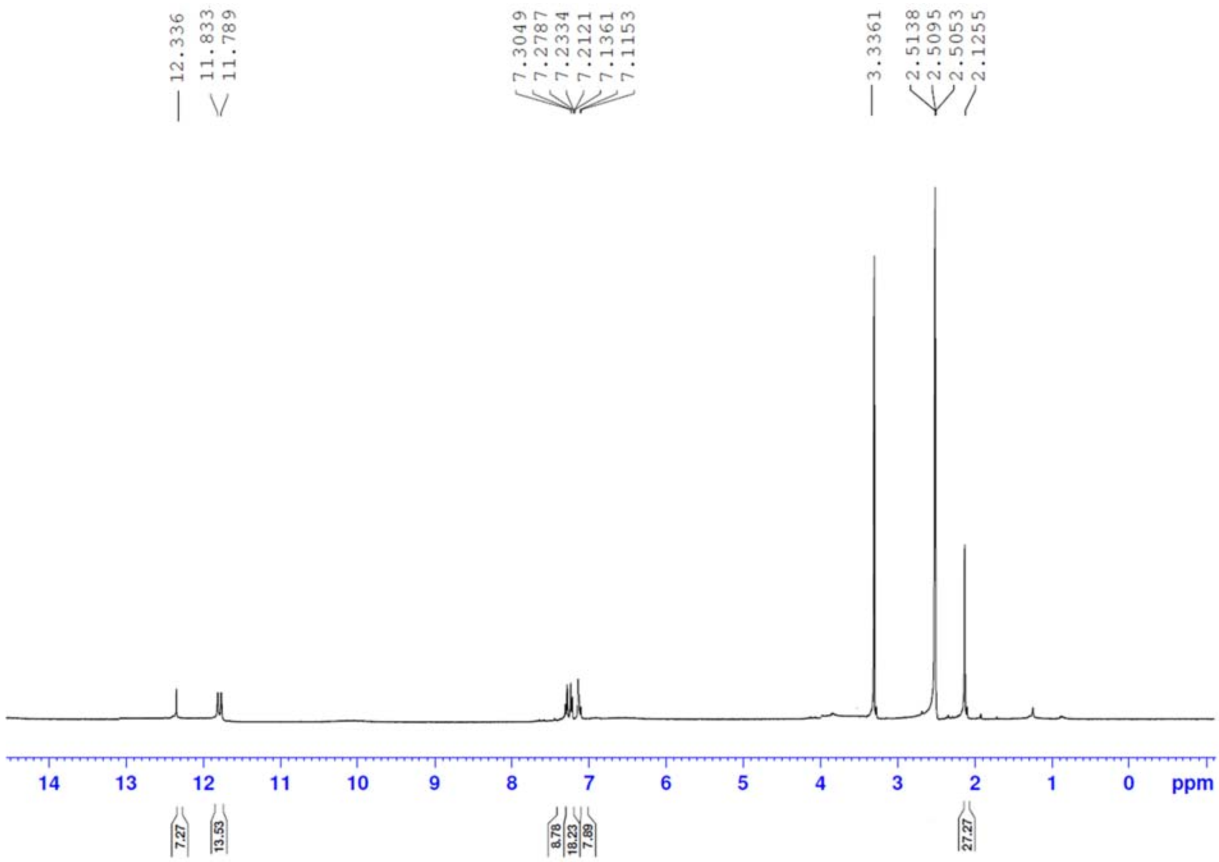

Figure S1. ^{1}H NMR spectrum of compound **4**

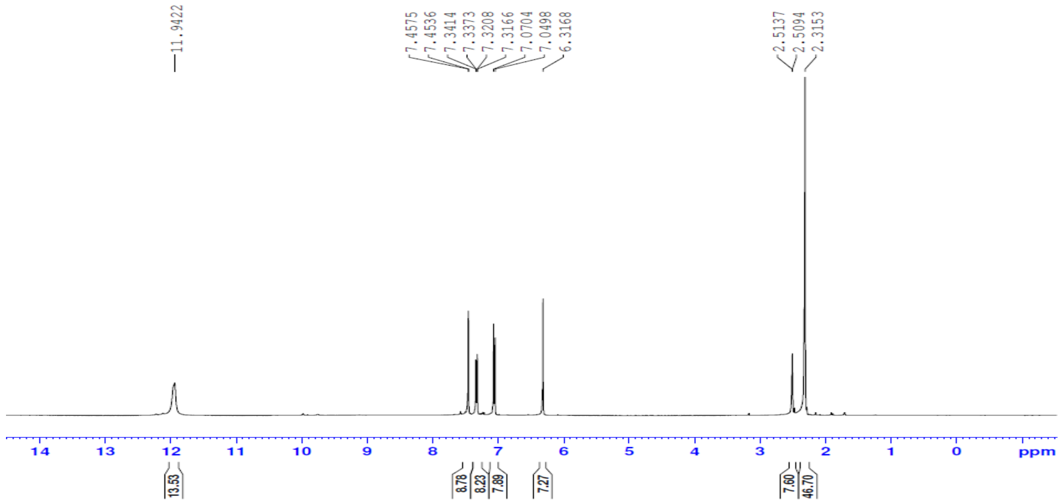

Figure S2. ^{1}H NMR spectrum of compound **5**

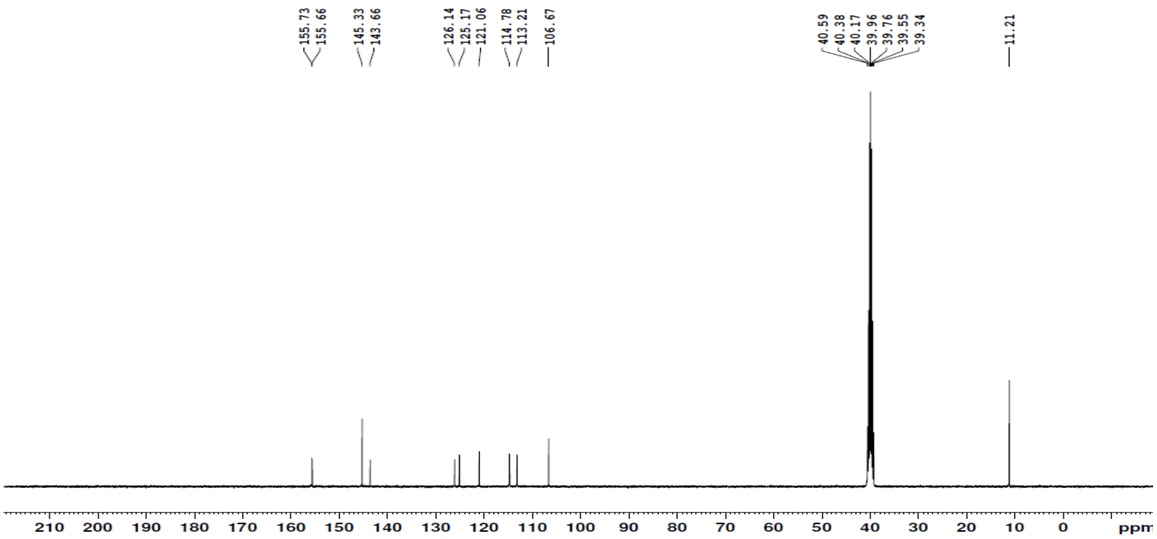

Figure S3. ^{13}C NMR spectrum of compound **5**

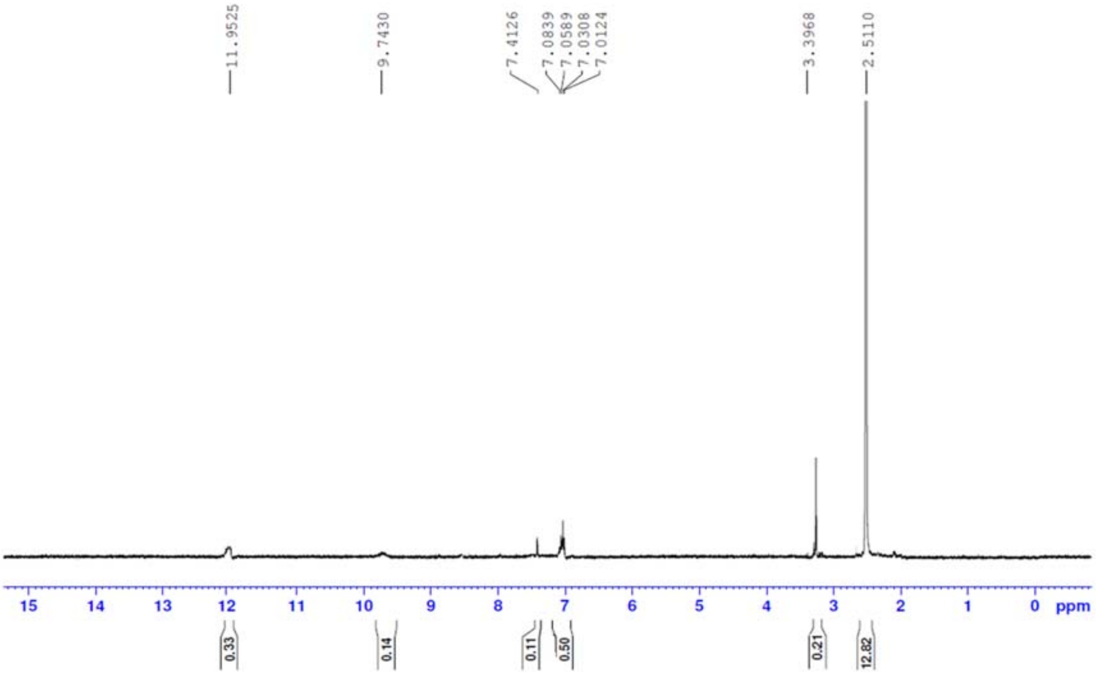

Figure S4. ^{1}H NMR spectrum of compound **6**

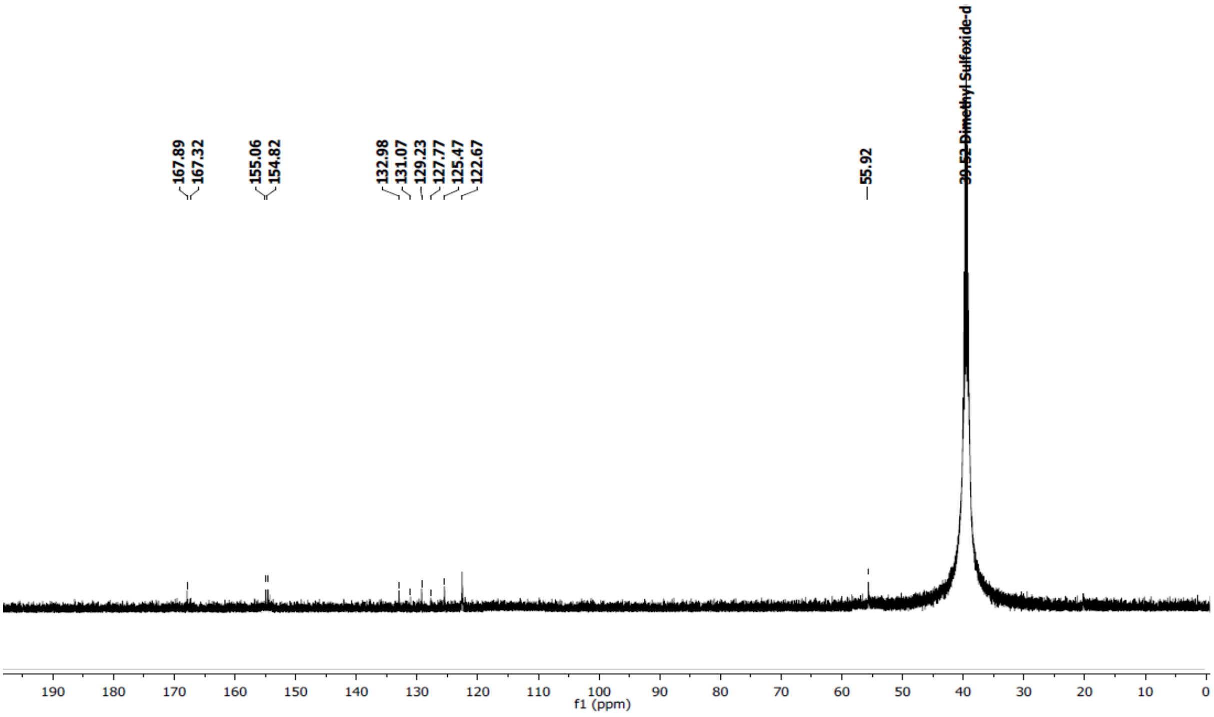

Figure S5. ^{13}C NMR spectrum of compound **6**

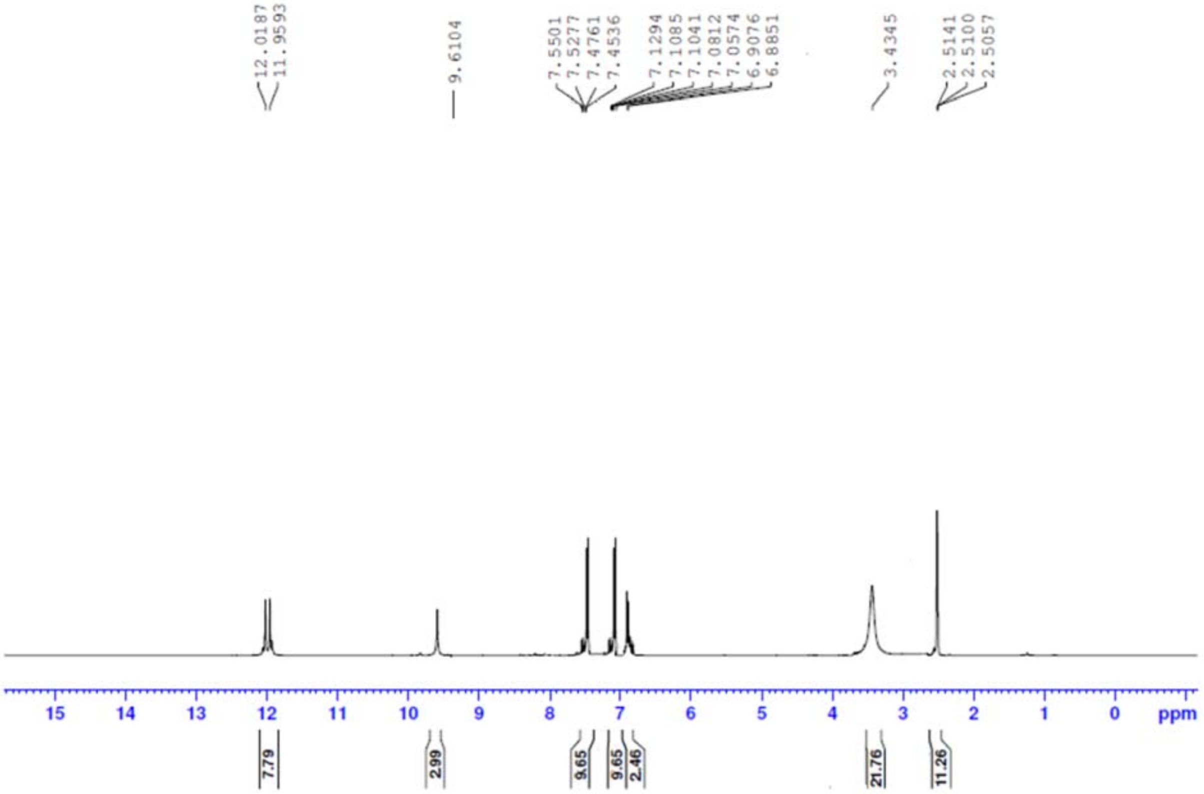

Figure S6. ^{1}H NMR spectrum of compound **7c**

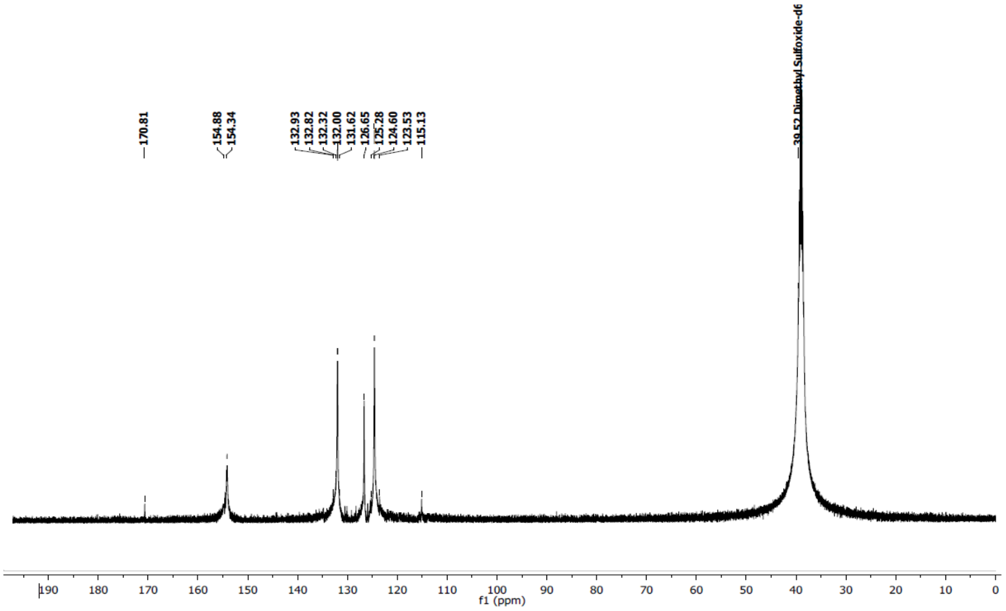

Figure S7. ^{13}C NMR spectrum of compound **7c**

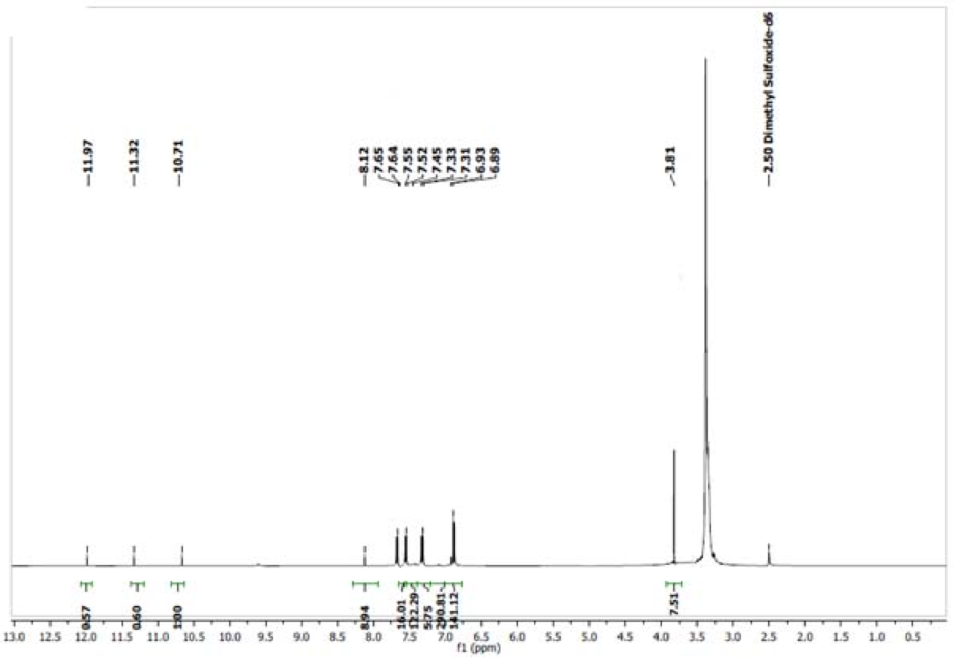

Figure S8. ^{1}H NMR spectrum of compound **8a**

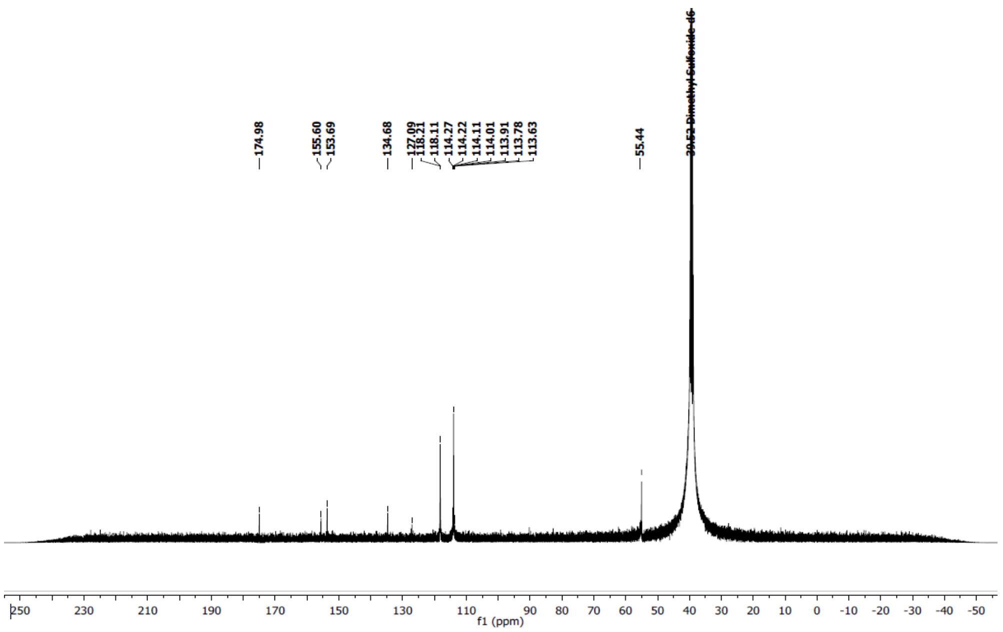

Figure S9. ^{13}C NMR spectrum of compound **8a**

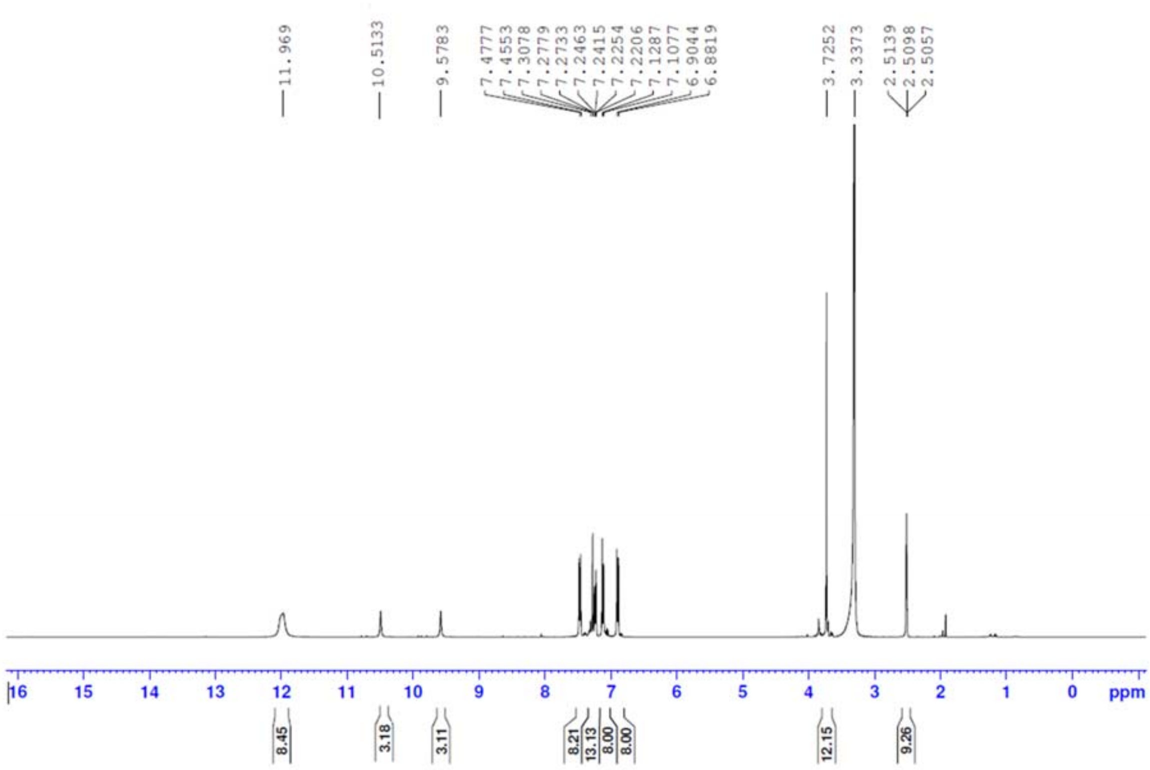

Figure S10 ^{1}H NMR spectrum of compound **9a**

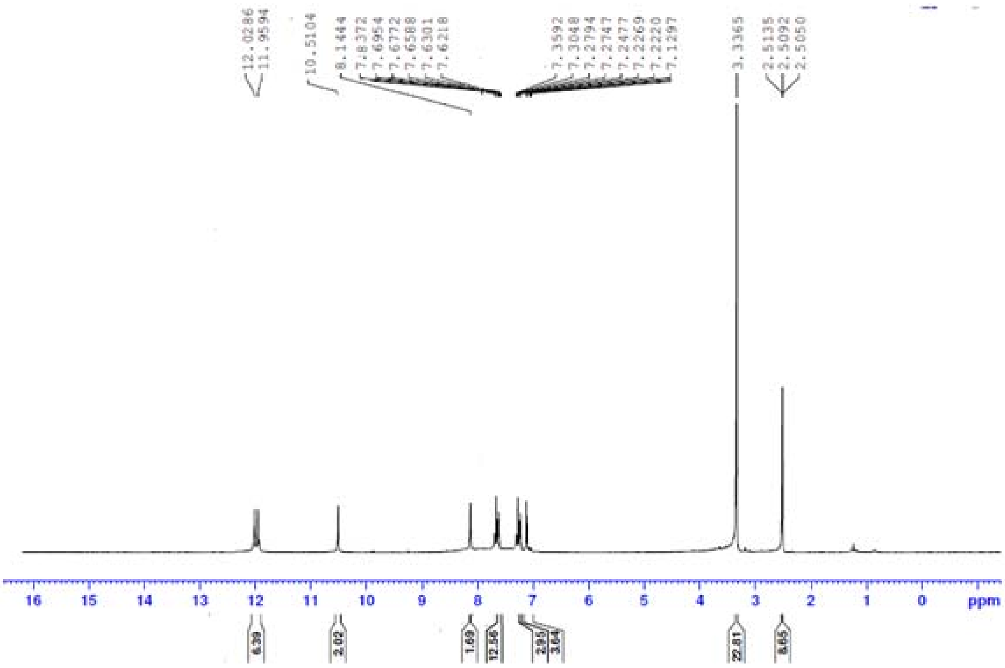

Figure S11. ^{1}HNMR spectrum of compound **9b**

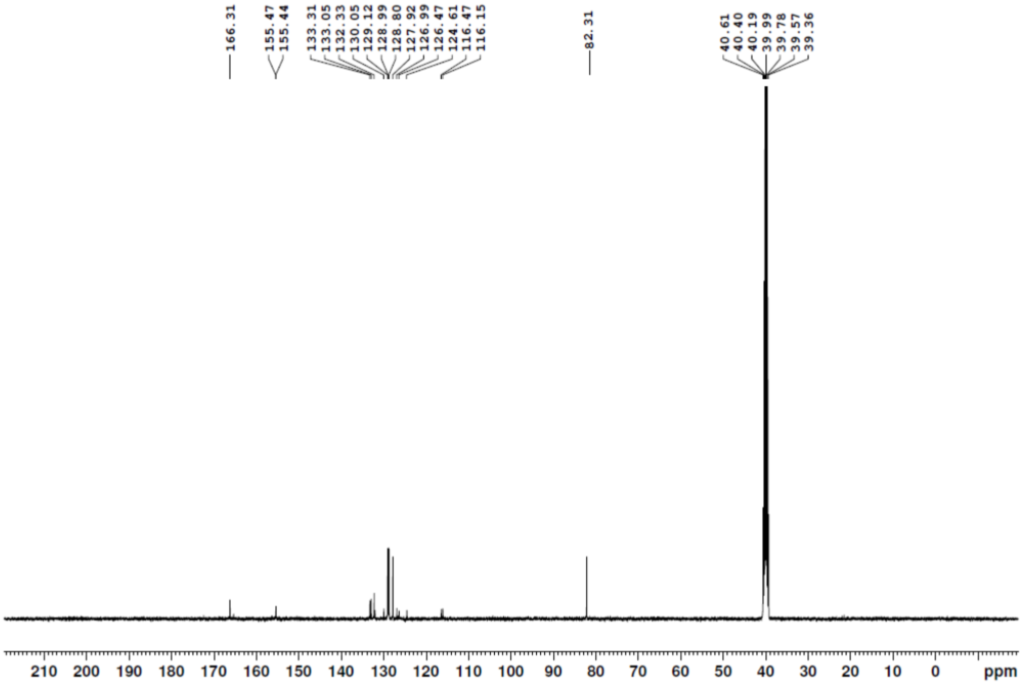

Figure S12. ^{13}C NMR spectrum of compound **9b**

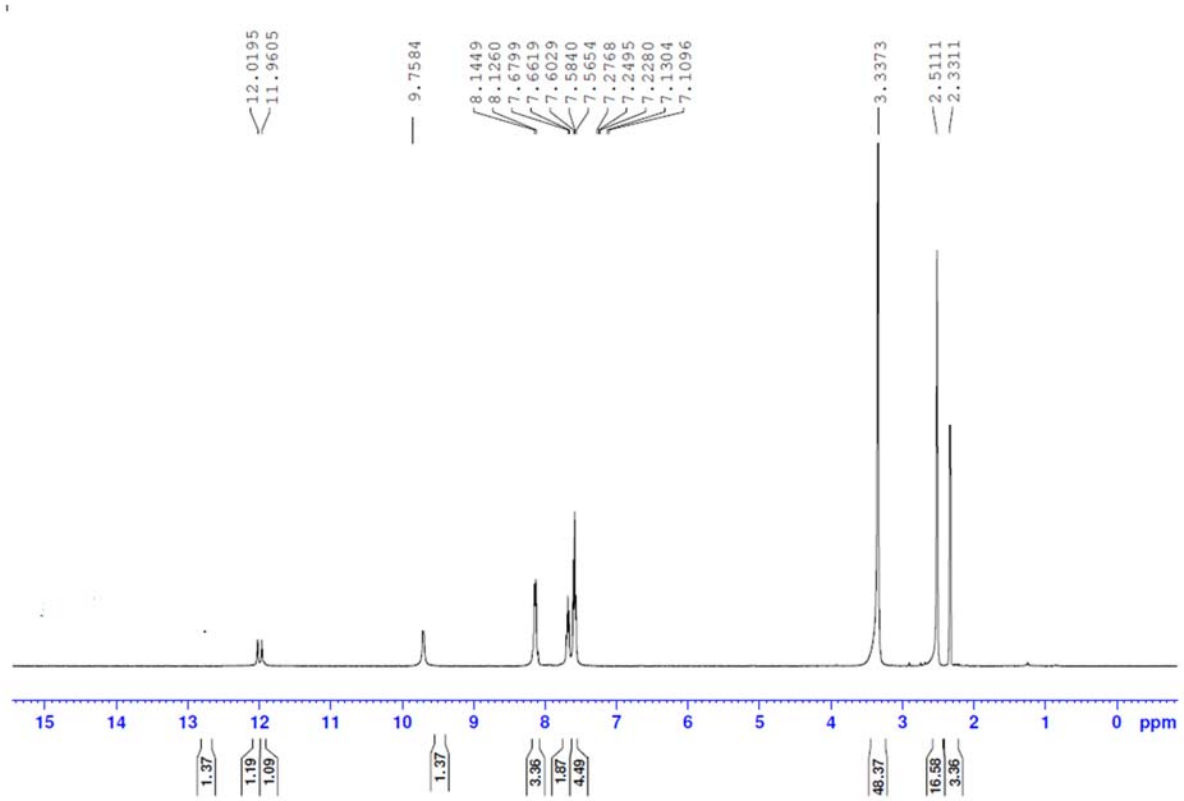

Figure S13. ^{1}H NMR spectrum of compound **10b**

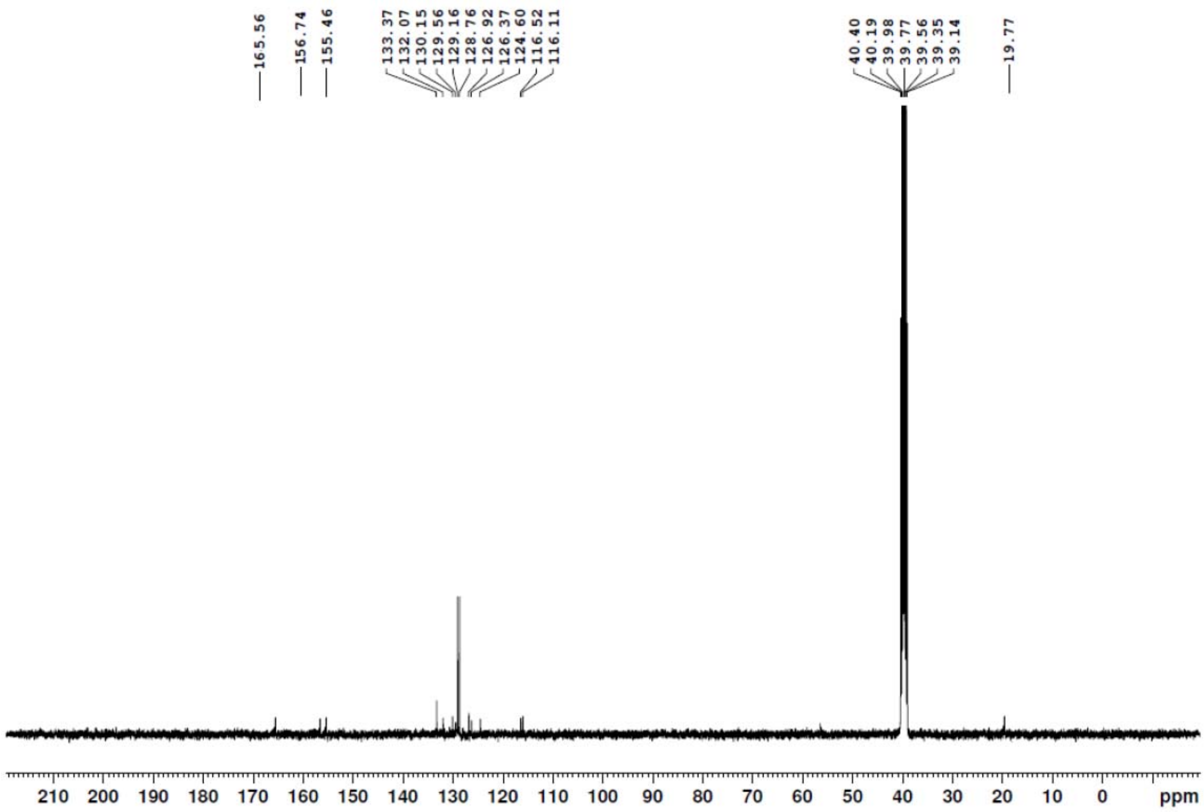

Figure S14. ^{13}C NMR spectrum of compound **10b**

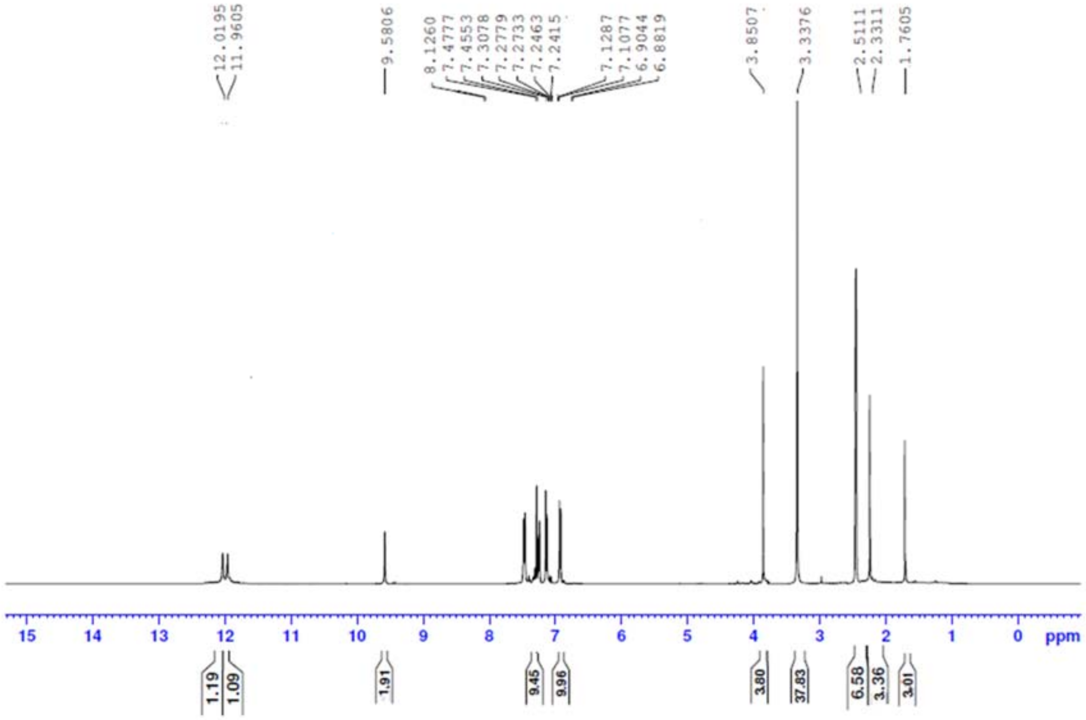

Figure S15. ^{1}H NMR spectrum of compound **11a**

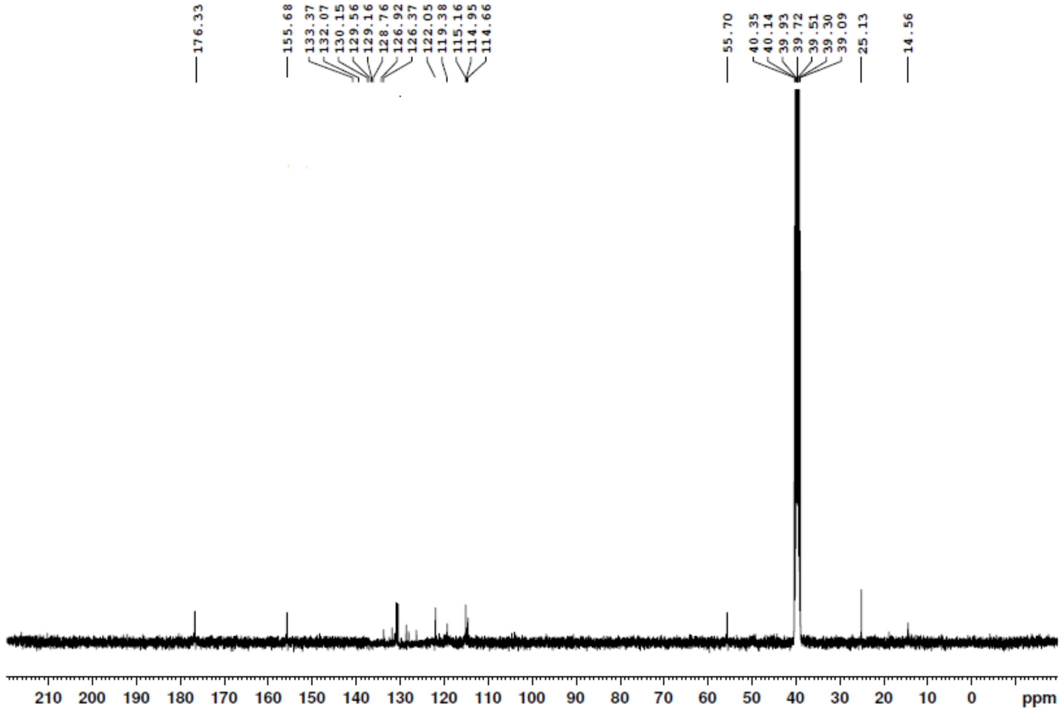

Figure S16. ^{13}C NMR spectrum of compound **11a**

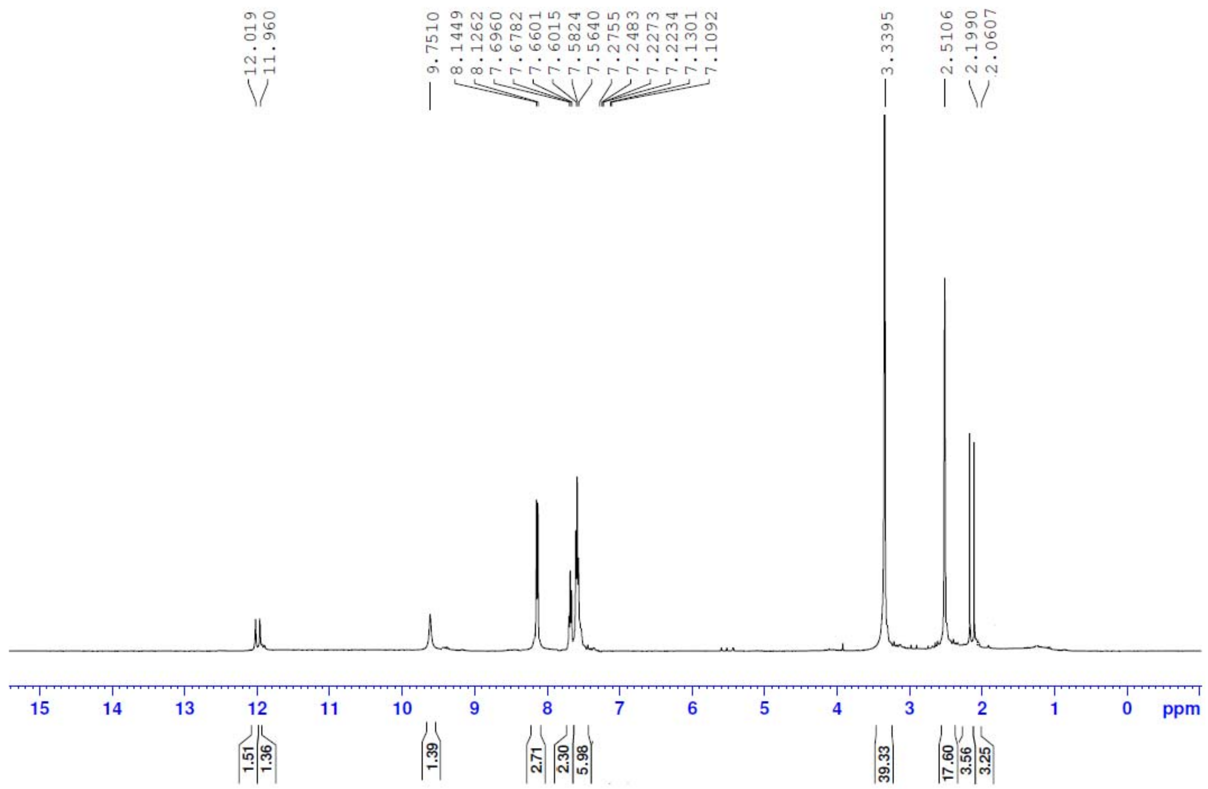

Figure S17. ^{1}H NMR spectrum of compound **11b**

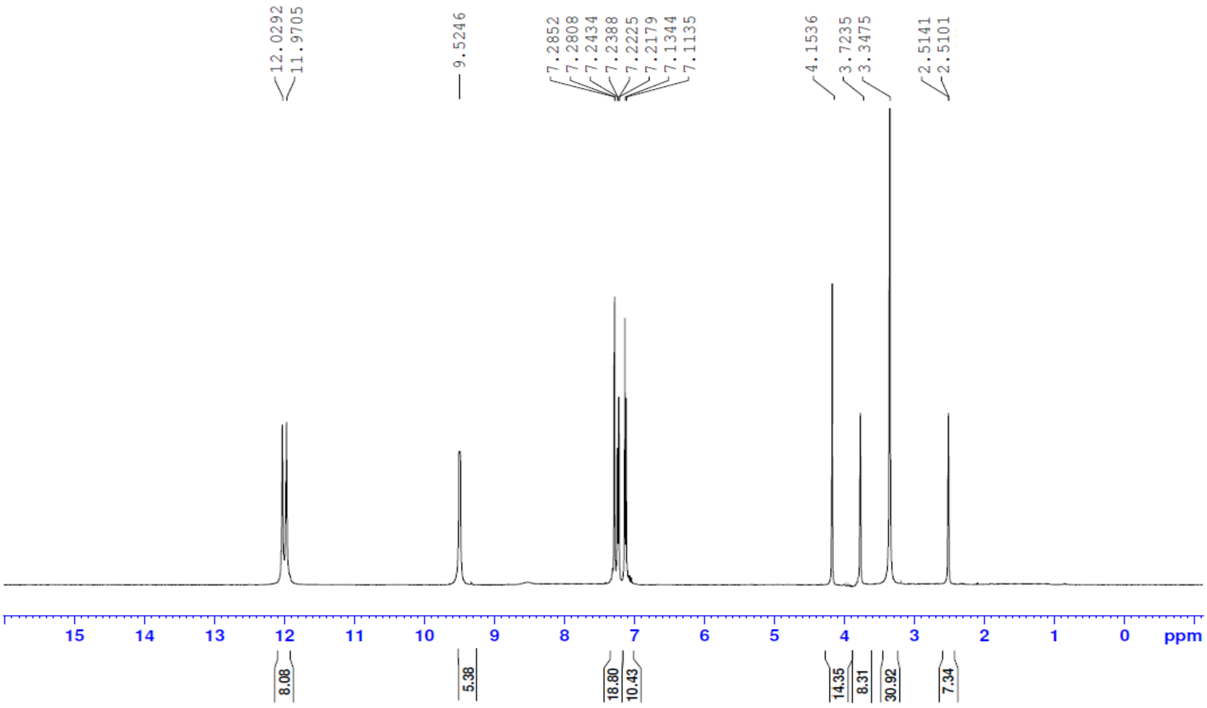

Figure S18. ^{1}H NMR spectrum of compound **12a**

Detailed results of PARP-1 enzyme assay

PARP1													

[redacted]

code	IC50	conc.ng/ml	log conc	%inh	T2	T1	ΔT	RFU2	RFU1	ΔRFU	slope	K.Activity	EC
1		10000	4	89.63	30	0	30	295	0	295	94.867	12.4385	120
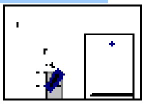		1000	3	80.25	30	0	30	562	0	562	94.867	23.6963	120
		100	2	59.87	30	0	30	1142	0	1142	94.867	48.1516	120
		10	1	49.54	30	0	30	1436	0	1436	94.867	60.5479	120
EC				0	30	0	30	2846	0	2846	94.867	120	120

[redacted]

code	IC50	conc.ng/ml	log conc	%inh	T2	T1	ΔT	RFU2	RFU1	ΔRFU	slope	K.Activity	EC
2		10000	4	89.92	30	0	30	287	0	287	94.867	12.1012	120
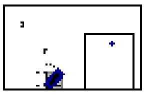		1000	3	80.71	30	0	30	549	0	549	94.867	23.1482	120
		100	2	67.92	30	0	30	913	0	913	94.867	38.496	120
		10	1	55.34	30	0	30	1271	0	1271	94.867	53.5908	120
EC				0	30	0	30	2846	0	2846	94.867	120	120

[redacted]

code	IC50	conc.ng/ml	log conc	%inh	T2	T1	ΔT	RFU2	RFU1	ΔRFU	slope	K.Activity	EC
3		10000	4	89.56	30	0	30	297	0	297	94.867	12.5228	120
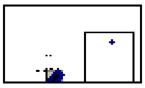		1000	3	76.04	30	0	30	682	0	682	94.867	28.756	120
		100	2	65.11	30	0	30	993	0	993	94.867	41.8691	120
		10	1	50.04	30	0	30	1422	0	1422	94.867	59.9576	120
EC				0	30	0	30	2846	0	2846	94.867	120	120

[redacted]

code	IC50	conc.ng/ml	log conc	%inh	T2	T1	ΔT	RFU2	RFU1	ΔRFU	slope	K.Activity	EC
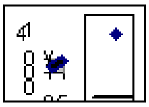		10000	4	88.79	30	0	30	319	0	319	94.867	13.4504	120
		1000	3	72.1	30	0	30	794	0	794	94.867	33.4784	120
		100	2	63.7	30	0	30	1033	0	1033	94.867	43.5557	120
		10	1	47.79	30	0	30	1486	0	1486	94.867	62.6561	120
EC				0	30	0	30	2846	0	2846	94.867	120	120

[redacted]

code	IC50	conc.ng/ml	log conc	%inh	T2	T1	ΔT	RFU2	RFU1	ΔRFU	slope	K.Activity	EC
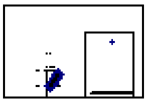		10000	4	86.58	30	0	30	382	0	382	94.867	16.1068	120
		1000	3	71.64	30	0	30	807	0	807	94.867	34.0266	120
		100	2	56.18	30	0	30	1247	0	1247	94.867	52.5789	120
		10	1	47.47	30	0	30	1495	0	1495	94.867	63.0356	120
EC				0	30	0	30	2846	0	2846	94.867	120	120

[redacted]

code	IC50	conc.ng/ml	log conc	%inh	T2	T1	ΔT	RFU2	RFU1	ΔRFU	slope	K.Activity	EC

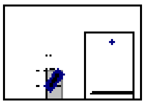		10000	4	89.88	30	0	30	288	0	288	94.867	12.1433	120
		1000	3	75.54	30	0	30	696	0	696	94.867	29.3463	120
		100	2	60.15	30	0	30	1134	0	1134	94.867	47.8143	120
		10	1	45.85	30	0	30	1541	0	1541	94.867	64.9752	120
EC				0	30	0	30	2846	0	2846	94.867	120	120

code	IC50	conc.ng/ml	log conc	%inh	T2	T1	ΔT	RFU2	RFU1	ΔRFU	slope	K.Activity	EC
**7**		10000	4	93.96	30	0	30	172	0	172	94.867	7.25226	120
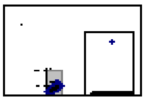													
		1000	3	87.67	30	0	30	351	0	351	94.867	14.7997	120
		100	2	73.12	30	0	30	765	0	765	94.867	32.2557	120
		10	1	55.69	30	0	30	1261	0	1261	94.867	53.1692	120
EC				0	30	0	30	2846	0	2846	94.867	120	120

code	IC50	conc.ng/ml	log conc	%inh	T2	T1	ΔT	RFU2	RFU1	ΔRFU	slope	K.Activity	EC
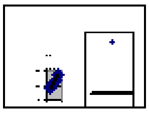		10000	4	86.23	30	0	30	392	0	392	94.867	16.5284	120
		1000	3	66.76	30	0	30	946	0	946	94.867	39.8874	120
		100	2	55.24	30	0	30	1274	0	1274	94.867	53.7173	120
		10	1	42.69	30	0	30	1631	0	1631	94.867	68.77	120
EC				0	30	0	30	2846	0	2846	94.867	120	120

code	IC50	conc.ng/ml	log conc	%inh	T2	T1	ΔT	RFU2	RFU1	ΔRFU	slope	K.Activity	EC
**9**		10000	4	87.46	30	0	30	357	0	357	94.867	15.0527	120
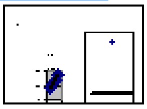													
		1000	3	67.85	30	0	30	915	0	915	94.867	38.5803	120
		100	2	52.07	30	0	30	1364	0	1364	94.867	57.5121	120
		10	1	40.55	30	0	30	1692	0	1692	94.867	71.342	120
EC				0	30	0	30	2846	0	2846	94.867	120	120

code	IC50	conc.ng/ml	log conc	%inh	T2	T1	ΔT	RFU2	RFU1	ΔRFU	slope	K.Activity	EC
**10**		10000	4	93.61	30	0	30	182	0	182	94.867	7.6739	120
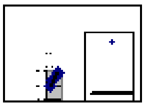													
		1000	3	80.99	30	0	30	541	0	541	94.867	22.8109	120
		100	2	65.36	30	0	30	986	0	986	94.867	41.574	120
		10	1	48.31	30	0	30	1471	0	1471	94.867	62.0237	120
EC				0	30	0	30	2846	0	2846	94.867	120	120

code	IC50	conc.ng/ml	log conc	%inh	T2	T1	ΔT	RFU2	RFU1	ΔRFU	slope	K.Activity	EC
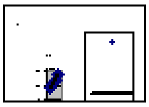		10000	4	86.23	30	0	30	392	0	392	94.867	16.5284	120
		1000	3	68.76	30	0	30	889	0	889	94.867	37.4841	120
		100	2	50.39	30	0	30	1412	0	1412	94.867	59.536	120
		10	1	40.69	30	0	30	1688	0	1688	94.867	71.1733	120
EC				0	30	0	30	2846	0	2846	94.867	120	120

code	IC50	conc.ng/ml	log conc	%inh	T2	T1	ΔT	RFU2	RFU1	ΔRFU	slope	K.Activity	EC

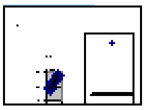

code	IC50	conc.ng/ml	log conc	%inh	T2	T1	ΔT	RFU2	RFU1	ΔRFU	slope	K.Activity	EC
		10000	4	88.19	30	0	30	336	0	336	94.867	14.1672	120
		1000	3	72	30	0	30	797	0	797	94.867	33.6049	120
		100	2	58.47	30	0	30	1182	0	1182	94.867	49.8382	120
		10	1	40.55	30	0	30	1692	0	1692	94.867	71.342	120
EC				0	30	0	30	2846	0	2846	94.867	120	120

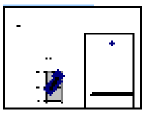

code	IC50	conc.ng/ml	log conc	%inh	T2	T1	ΔT	RFU2	RFU1	ΔRFU	slope	K.Activity	EC
		10000	4	84.82	30	0	30	432	0	432	94.867	18.215	120
		1000	3	66.55	30	0	30	952	0	952	94.867	40.1404	120
		100	2	51.69	30	0	30	1375	0	1375	94.867	57.9759	120
		10	1	39.42	30	0	30	1724	0	1724	94.867	72.6912	120
EC				0	30	0	30	2846	0	2846	94.867	120	120

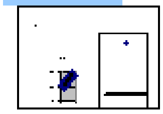

code	IC50	conc.ng/ml	log conc	%inh	T2	T1	ΔT	RFU2	RFU1	ΔRFU	slope	K.Activity	EC
14		10000	4	89.63	30	0	30	295	0	295	94.867	12.4385	120
		1000	3	78.25	30	0	30	619	0	619	94.867	26.0997	120
		100	2	63.88	30	0	30	1028	0	1028	94.867	43.3449	120
		10	1	50.91	30	0	30	1397	0	1397	94.867	58.9035	120
EC				0	30	0	30	2846	0	2846	94.867	120	120

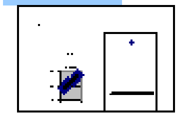

code	IC50	conc.ng/ml	log conc	%inh	T2	T1	ΔT	RFU2	RFU1	ΔRFU	slope	K.Activity	EC
15		10000	4	88.09	30	0	30	339	0	339	94.867	14.2937	120
		1000	3	67.57	30	0	30	923	0	923	94.867	38.9176	120
		100	2	56.82	30	0	30	1229	0	1229	94.867	51.8199	120
		10	1	42.8	30	0	30	1628	0	1628	94.867	68.6435	120
EC				0	30	0	30	2846	0	2846	94.867	120	120

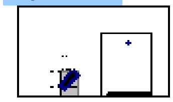

code	IC50	conc.ng/ml	log conc	%inh	T2	T1	ΔT	RFU2	RFU1	ΔRFU	slope	K.Activity	EC
16		10000	4	89.04	30	0	30	312	0	312	94.867	13.1553	120
		1000	3	79.13	30	0	30	594	0	594	94.867	25.0456	120
		100	2	65.53	30	0	30	981	0	981	94.867	41.3632	120
		10	1	51.65	30	0	30	1376	0	1376	94.867	58.0181	120
EC				0	30	0	30	2846	0	2846	94.867	120	120

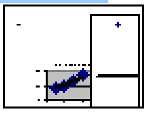

code	IC50	conc.ng/ml	log conc	%inh	T2	T1	ΔT	RFU2	RFU1	ΔRFU	slope	K.Activity	EC
17		10000	4	84.47	30	0	30	442	0	442	94.867	18.6366	120
		1000	3	66.3	30	0	30	959	0	959	94.867	40.4356	120
		100	2	49.86	30	0	30	1427	0	1427	94.867	60.1684	120
		10	1	40.55	30	0	30	1692	0	1692	94.867	71.342	120
EC				0	30	0	30	2846	0	2846	94.867	120	120

code	IC50	conc.ng/ml	log conc	%inh	T2	T1	ΔT	RFU2	RFU1	ΔRFU	slope	K.Activity	EC
**18**		10000	4	86.47	30	0	30	385	0	385	94.867	16.2333	120
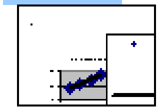		1000	3	69.04	30	0	30	881	0	881	94.867	37.1467	120
		100	2	55.41	30	0	30	1269	0	1269	94.867	53.5065	120
		10	1	41.78	30	0	30	1657	0	1657	94.867	69.8662	120
EC				0	30	0	30	2846	0	2846	94.867	120	120

code	IC50	conc.ng/ml	log conc	%inh	T2	T1	ΔT	RFU2	RFU1	ΔRFU	slope	K.Activity	EC
**olaparib**		10000	4	93.64	30	0	30	181	0	181	94.867	7.63174	120
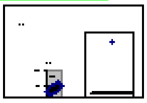		1000	3	86.16	30	0	30	394	0	394	94.867	16.6127	120
		100	2	66.37	30	0	30	957	0	957	94.867	40.3512	120
		10	1	55.27	30	0	30	1273	0	1273	94.867	53.6751	120
EC				0	30	0	30	2846	0	2846	94.867	120	120

Lab Report

*IC50 results

researcher	assay	Date	cells
	MTT	03-Nov	MDA-MB-436

	**Blank**	**CC**	**Sample No.**		**10b/MDA-MB-436**			**Sample No.**		**4/MDA-MB-436**		
	**1**	**2**	**3**	**4**	**5**	**6**	**7**	**8**	**9**	**10**	**11**	**12**
A	B	C	100uM	25uM	6.3uM	1.6uM	0.4uM	100uM	25uM	6.3uM	1.6uM	0.4uM
B	B	C	100uM	25uM	6.3uM	1.6uM	0.4uM	100uM	25uM	6.3uM	1.6uM	0.4uM
C	B	C	100uM	25uM	6.3uM	1.6uM	0.4uM	100uM	25uM	6.3uM	1.6uM	0.4uM

ROBONIK P2000 Eia reader

Wave length: 450 nm

Reference: 630 nm

	**1**	**2**	**3**	**4**	**5**	**6**	**7**	**8**	**9**	**10**	**11**	**12**
A	0.001	0.539	0.181	0.238	0.289	0.348	0.393	0.237	0.278	0.331	0.383	0.451
B	0.001	0.557	0.169	0.245	0.295	0.356	0.404	0.223	0.287	0.33	0.385	0.444
C	0.001	0.544	0.181	0.227	0.275	0.366	0.395	0.229	0.285	0.325	0.376	0.447
mean	4E-04	0.547	0.177	0.2367	0.2863	0.3567	0.3973	0.23	0.283	0.329	0.3813	0.447
%		`	32.38	43.293	52.378	65.244	72.683	42.01	51.83	60.12	69.756	81.83

10b/MDA-MB-436

log conc.	% viability
2	32.38
1.398	43.29
0.796	52.38
0.193	65.24
-0.409	72.68

4/MDA-MB-436

log conc.	% viability
2	42.01
1.398	51.83
0.796	60.12
0.193	69.76
-0.409	81.83

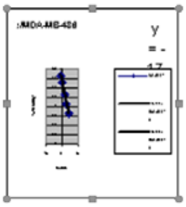

IC50=

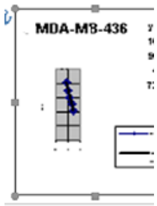

IC50=

	Blank	CC	Sample No.		8a/MDA-MB-436			Sample No.		5/MDA-MB-436		
	1	2	3	4	5	6	7	8	9	10	11	12
A	B	C	100uM	25uM	6.3uM	1.6uM	0.4uM	100uM	25uM	6.3uM	1.6uM	0.4uM
B	B	C	100uM	25uM	6.3uM	1.6uM	0.4uM	100uM	25uM	6.3uM	1.6uM	0.4uM
C	B	C	100uM	25uM	6.3uM	1.6uM	0.4uM	100uM	25uM	6.3uM	1.6uM	0.4uM

ROBONIK P2000 Eia reader

Wave length: 450 nm

Reference: 630 nm

	1	2	3	4	5	6	7	8	9	10	11	12
A	0.001	0.575	0.216	0.259	0.313	0.364	0.424	0.166	0.222	0.258	0.318	0.354
B	0.001	0.581	0.197	0.261	0.329	0.349	0.419	0.159	0.208	0.261	0.312	0.357
C	0.001	0.593	0.213	0.248	0.294	0.358	0.441	0.171	0.218	0.271	0.305	0.343
mean	0.001	0.583	0.209	0.256	0.312	0.357	0.428	0.165	0.216	0.263	0.3117	0.351
% viability			35.79	43.911	53.516	61.235	73.413	28.36	37.05	45.17	53.459	60.26

8a/MDA-MB-436

log conc.	% viability
2	35.79
1.398	43.91
0.796	53.52
0.193	61.23
-0.409	73.41

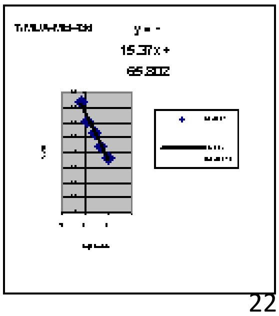

5/MDA-MB-436

log conc.	% viability
2	28.36
1.398	37.05
0.796	45.17
0.193	53.46
-0.409	60.26

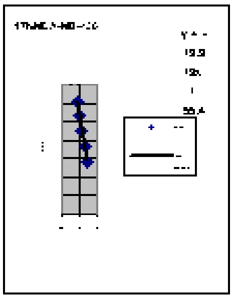

IC50=		IC50=	

	Blank	CC	Sample No.		18b/MDA-MB-436			Sample No.		STA/MDA-MB-436		
	1	2	3	4	5	6	7	8	9	10	11	12
A	B	C	100uM	25uM	6.3uM	1.6uM	0.4uM	100uM	25uM	6.3uM	1.6uM	0.4uM
B	B	C	100uM	25uM	6.3uM	1.6uM	0.4uM	100uM	25uM	6.3uM	1.6uM	0.4uM
C	B	C	100uM	25uM	6.3uM	1.6uM	0.4uM	100uM	25uM	6.3uM	1.6uM	0.4uM

ROBONIK P2000 Eia reader

Wave length: 450 nm

Reference: 630 nm

	1	2	3	4	5	6	7	8	9	10	11	12
A	0.001	0.549	0.188	0.251	0.285	0.352	0.421	0.182	0.241	0.292	0.348	0.412
B	0.003	0.552	0.192	0.241	0.313	0.337	0.395	0.175	0.244	0.278	0.351	0.388
C	0.001	0.561	0.206	0.252	0.318	0.342	0.405	0.181	0.239	0.291	0.347	0.395
mean	0.002	0.554	0.195	0.248	0.3053	0.3437	0.407	0.179	0.241	0.287	0.3487	0.398
% viability			35.26	44.765	55.114	62.034	73.466	32.37	43.56	51.81	62.936	71.9

11b/MDA-MB-436

2	35.26
1.398	44.77
0.796	55.11
0.193	62.03
-0.409	73.47

STA/MDA-MB-436

2	32.37
1.398	43.56
0.796	51.81
0.193	62.94
-0.409	71.9

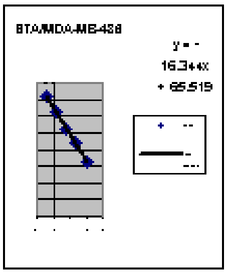

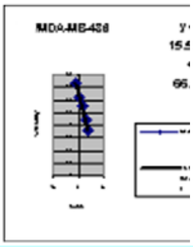

IC50=		IC50=	
